# A Clinical Study of the Intra-Neuroendoscopic Technique for the Treatment of Subacute-Chronic and Chronic Septal Subdural Hematoma

**DOI:** 10.3389/fneur.2019.01408

**Published:** 2020-01-17

**Authors:** Bo Du, Jianzhong Xu, Jintao Hu, Xianliang Zhong, Jian Liang, Pengfei Lei, Hao Wang, Weichun Li, Yuping Peng, Aijun Shan, Yujuan Zhang

**Affiliations:** ^1^Department of Emergency, Shenzhen People's Hospital, The Second Clinical Medical College of Jinan University, The First Affiliated Hospital of Southern University of Science and Technology, Shenzhen, China; ^2^Department of Pathology, Shenzhen People's Hospital, The Second Clinical Medical College of Jinan University, The First Affiliated Hospital of Southern University of Science and Technology, Shenzhen, China; ^3^Department of Neurosurgery, Shenzhen People's Hospital, The Second Clinical Medical College of Jinan University, The First Affiliated Hospital of Southern University of Science and Technology, Shenzhen, China; ^4^Department of Neurosurgery, Nanfang Hospital, Southern Medical University, Guangzhou, China; ^5^Department of Ultrasound, Shenzhen People's Hospital, The Second Clinical Medical College of Jinan University, The First Affiliated Hospital of Southern University of Science and Technology, Shenzhen, China

**Keywords:** intra-neuroendoscopic technique (INET), transparent sheath, chronic subdural hematoma (CSDH), inflammatory factor, pathology, prognosis

## Abstract

**Objective:** The surgical technique, safety, efficacy, and clinical application value of the intra-neuroendoscopic technique (INET) for the treatment of subacute-chronic and chronic septal subdural hematoma was investigated based on the structure and pathological features of the hematoma wall, and the critical factors of hematoma growth and recurrence were determined, in order to provide reference for clinical drug treatment.

**Methods:** This was non-randomized concurrent control study. A total of 94 patients who met the inclusion criteria were recruited between May 2015 and February 2019 and were divided into the INET treatment group (INET group, 45 cases) and the burr hole drainage (BHD) treatment group (control group, 49 cases). The hematoma fluid components and the morphological structure and pathological characteristics of the hematoma wall were analyzed, and the surgical duration, subdural drainage tube (SDT) placement duration, intracranial infection rate, Bender grade at the 1 month post-operative follow-up and hematoma recurrence rate within the 6 months of post-operative follow-up were compared between the two groups. A multiple logistic regression model was established to analyze the risk factors associated with recurrence within 6 months.

**Results:** Intraoperative endoscopy showed that the adhesion bands that formed early in the hematoma cavity were strip-like and that those that formed late were lock-column-like. The hematoma cavity was divided into different-sized chambers with by these strips/columns. Pathological sections of cyst wall reveled angiogenesis inside the cyst and mucus-like changes, rupture and hemorrhage in the vascular wall. Obvious inflammatory cell infiltration and fibrous connective tissue hyperplasia were observed in the cyst wall. The osmotic pressure of the hematoma fluid was not significantly different from that of the peripheral venous blood [(296.7 ± 10.3) mOsm/kg vs. (291.5 ± 12.4) mOsm/kg, *p* = 0.68]. However, the D-dimer contents which reflect the severity of fibrinolysis in the hematoma and the proinflammatory cytokine interleukin 6 (IL-6) were significantly higher in the hematoma fluid than in the peripheral venous blood. The surgery duration for the INET group was significantly longer than that for the control group [(60.4 ± 10.6) min vs. (44.1 ± 9.8) min, *p* = 0.00], but both the hematoma recurrence rate within 6 months of post-operative follow-up (4.4 vs. 24.5%, *p* = 0.00) and the SDT placement duration [(2.1 ± 0.6) d vs. (3.9 ± 0.7) d, *p* = 0.00] for the INET group were both lower than those for the control group. The intracranial infection rate did not differ significantly between the two groups (4.4 vs. 10.2%, *p* = 0.50). The overall effective rate of the Bender grade at 1 month of follow-up did not differ significantly between the two groups (95.6 vs. 87.8%, *p* = 0.32), but the proportion of patients who recovered to Bender grade 0 with no symptoms was significantly higher in the INET group than in the control group (86.7 vs. 67.3%, *p* = 0.03). Multiple logistic regression analysis showed that INET surgery [odds ratio (OR) 3.71, 95% confidence interval (CI) 1.31–9.62, *p* = 0.02], age of 65 years or younger (OR 1.51, 95% CI 1.05–2.87, *p* = 0.03) and unilateral subdural hematoma (OR 1.76, 95% CI 1.05–3.41, *p* = 0.02) were independent factors that reduced the post-operative recurrence rate.

**Conclusion:** The INET surgical plan based on the structure and pathological features of the subacute-chronic and chronic subdural hematoma wall can reduce the recurrence rate and improve the clinical prognosis.

**Trial registration:** ClinicalTrials.gov, NCT02515903. Registered 5 August, 2015.

—*a surgical treatment plan based on the structure and pathological features of the subacute-chronic and chronic subdural hematoma wall that reduces the recurrence rate and improves patient prognosis*

## Introduction

Chronic subdural hematoma (cSDH) often affects middle-aged and elderly individuals with a history of minor trauma and is a common intracranial hemorrhagic disease, accounting for approximately 10% of all intracranial hematomas. Currently, the most preferred treatment for cSDH is still unclear (1). The incidence of cSDH has been increasing. According to the latest reports (2–4), the incidence of cSHD among individuals older than 65 years has increased from 8–18/100,000 15 years ago to 48/100,000. The recurrence rate of hematoma after different surgeries in patients with cSDH is 2.3–38.7% (3, 5–8). Burr hole drainage (BHD) is still a classic surgical procedure for treating cSDH and is a simple and effective method for alleviating early brain compression symptoms. However, the rate of patients requiring reoperation due to post-operative recurrence is as high as 31.6% (3). Subacute-chronic and chronic septal subdural hematomas are complicated cSDH types with high post-operative recurrence rates. Because a large number of recurrent patients are elderly and often accompanied with multiple organ or systemic disease, they are unable to tolerate highly traumatic craniotomy, which makes these recurrent cases difficult to treat clinically. Based on the structure and pathological features of the subacute-chronic and chronic septal subdural hematoma wall, the intra-neuroendoscopic technique (INET) was performed in this non-randomized concurrent control study (classic BHD was the control treatment), and the surgical technique, safety, efficacy, and clinical value of INET for the treatment of these diseases were investigated. The key factors of hematoma growth and recurrence were analyzed through clinical and pathological examinations, aiming to provide a reference for the clinical treatment of cSDH.

## Materials and Methods

### General Data

A total of 94 patients who met the inclusion criteria were recruited between May 2015 and February 2019 from Shenzhen People's Hospital and Nanfang Hospital. The patients were divided into two groups according to the preferences of the patients and their families: 45 patients in the INET group and 49 patients in the control group. The two groups did not differ significantly in sex, average age, midline shift, pre-operative hematoma size, pre-operative Bender grade, and medical history (such as definitive trauma, anticoagulant drugs, hypertension, diabetes, and stroke) (*P* > 0.05); therefore, the two groups were comparable. However, the two groups differed significantly in the proportions of patients aged 65 years and younger and patients who had a unilateral subdural hematoma (*P* < 0.05) (Table 1).

**Table 1:** Comparison of pre-operative baseline data between the INET and control groups.

**Baseline indicator**	**INET group** ** (*n* = 45)**	**Control group** ** (*n* = 49)**	***p*-value**
Sex (Male/Female)	29/16	30/19	0.75
Age (Years)	73.2 ± 5.5	70.6 ± 6.1	0.17
Age ≤65 years [n(%)]	9 (20.0%)	20 (40.8%)	0.03
A definitive history of trauma [n(%)]	37 (82.2%)	34 (69.4%)	0.15
Unilateral subdural hemorrhage [n(%)]	38 (84.4%)	32 (65.3%)	0.03
Midline shift (mm)	9.6 ± 3.1	8.8 ± 3.8	0.52
Hematoma volume (ml)	96.8 ± 19.2	104.3 ± 21.3	0.36
Use of anticoagulant/antiplatelet drugs [*n* (%)]	23 (51.1%)	30 (61.2%)	0.32
Hypertension	32 (71.1%)	28 (57.1%)	0.16
Diabetes	22 (48.9%)	30 (61.2%)	0.23
Stroke	17 (37.8%)	14 (28.6%)	0.47
**Bender grade**			
Grade I	9 (20.0%)	15 (30.6%)	0.24
Grade II	24 (53.3%)	26 (53.1%)	0.67
Grade III	12 (26.7%)	8 (16.3%)	0.22

All patients underwent cranial computed tomography (CT) to confirm the diagnosis. The hematoma volume was measured according to the Coniglobus formula (9), and the severity of the midline shift was determined by two senior physicians who averaged the measurements of the layer with the maximum midline shift on CT images. This study was approved by the ethics committee of the hospital (approval No. NFEC-2015-034) and was registered as clinical research at ClinicalTrials.gov (NCT 02515903).

### Case Inclusion and Exclusion Criteria

#### Inclusion Criteria

(1) Adults aged <85 years, (2) patients with clear clinical symptoms but were not comatose and did not have dilated pupils, and their Bender grade was I-III, (3) CT upon admission showed that the patients had subacute-chronic subdural hematoma, or the imaging revealed chronic septal subdural hematoma, compressed brain tissue and shifted midline structure, and (4) the patients or their family agreed to enter the clinical study and allowed follow-up observations.

#### Exclusion Criteria

(1) CT upon admission showed that the patients had acute subdural hematoma, (2) patients with severe systemic diseases, such as severe dysfunctions of the heart, liver, lung and kidney, and (3) patients with abnormal blood coagulation, such as long-term use of anticoagulant drugs or coagulopathy caused by abnormal blood coagulation.

### Research Methods and Observation Indicators

This was a non-randomized concurrent control study. There were three experienced doctors in our center to perform this operation, they have all undergone rigorous training. The investigators thoroughly explained the advantages and disadvantages, surgical risks, remedial measures, treatment costs and potential prognostic outcomes of the two techniques to each subject who met the inclusion and exclusion criteria. The patients and their family chose to join either group according to their preferences. After enrollment, the scalp incision length, bone hole diameter, surgery duration, subdural drainage tube (SDT) placement duration, intracranial infection rate during hospitalization, Bender grade of the patients at the 1 month post-operative follow-up and hematoma recurrence rate at the 6 month post-operative follow-up were compared between the two groups. For patients in the INET group, the hematoma cyst wall was taken during the operation for pathological examination, and the hematoma fluid and peripheral venous blood were taken to compare the osmotic pressure and IL-6 and D-dimer levels. During the study, patients were given 20 mg of atorvastatin orally, once a day for 3 months. The Bender grade criteria were as follows: Grade 0—no symptoms; Grade I—general symptoms, such as dizziness and headache, no unconsciousness, no psychiatric symptoms, and no obvious focal neurological deficits; Grade II—lethargy or confusion, psychiatric symptoms, and mild focal neurological deficits; Grade III—stupor, obvious psychiatric symptoms and focal neurological deficits; and Grade IV—coma or signs of herniation.

### INET

#### Instruments

**Figure 1:**
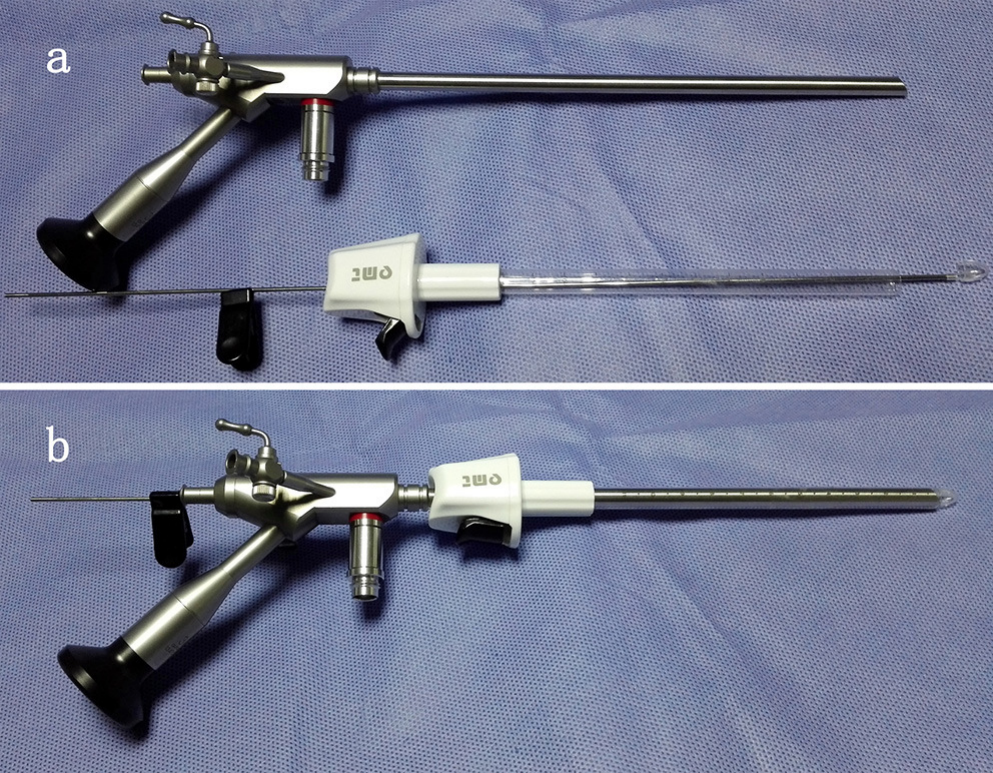
Transparent sheath and neuroendoscope before **(a)** and after **(b)** assembly.

The INET equipment in this study was composed of a high-definition imaging system, cold light source, Zeppelin large working channel endoscope, transparent sheath, hematoma smashing aspirator, endoscopic scissors, endoscopic tweezers, endoscopy biopsy forceps, and endoscope specialized bipolar electrocoagulator. The large working channel endoscope (NEH 0/30-177-6.5) had a working length of 177 mm, external diameter of 6.5 mm, and view angle of 0 or 30°. The working channel diameter was 3.7 mm, and the diameters of the two suction/flushing channels were both 1.5 mm (10–12). The application of INET relies on a novel product developed by our team-the transparent sheath (Chinese Patent No. ZL 200820046232.0, State Intellectual Property Office of P.R. China, website: http://cpquery.sipo.gov.cn), which can fit seamlessly on a Zeppelin large channel endoscope. The transparent sheath has an external diameter of 7.7 mm, is colorless and transparent and has a transparent tip that can be removed together with the endoscope as well as a scale and a fixation device (Figure 1).

#### Surgical Method

**Figure 2:**
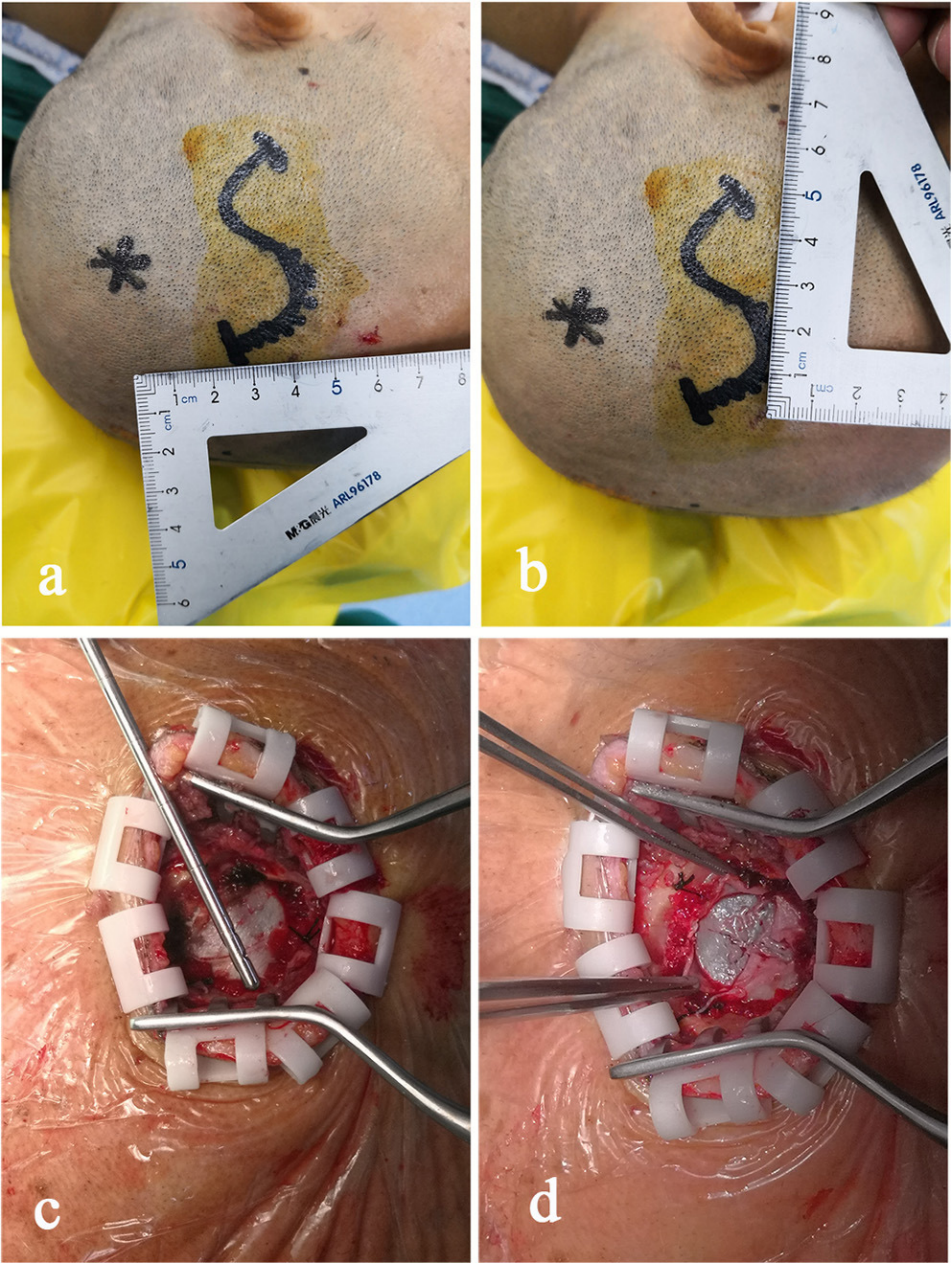
The midpoint of the surgical incision is generally 3–4 cm in front of the apical nodule. The direction of the incision is parallel to the scalp vessel, and the incision is in an “S” shape with a length of 4–5 cm **(a,b)**. After a hole is drilled in the skull, a small bone flap with a diameter of 2.0–3.0 cm is cut using a milling cutter **(c)**. The wall envelop of the hematoma sac can be seen using a cross-shaped incision after lifting the dura mater **(d)**.

**Figure 3:**
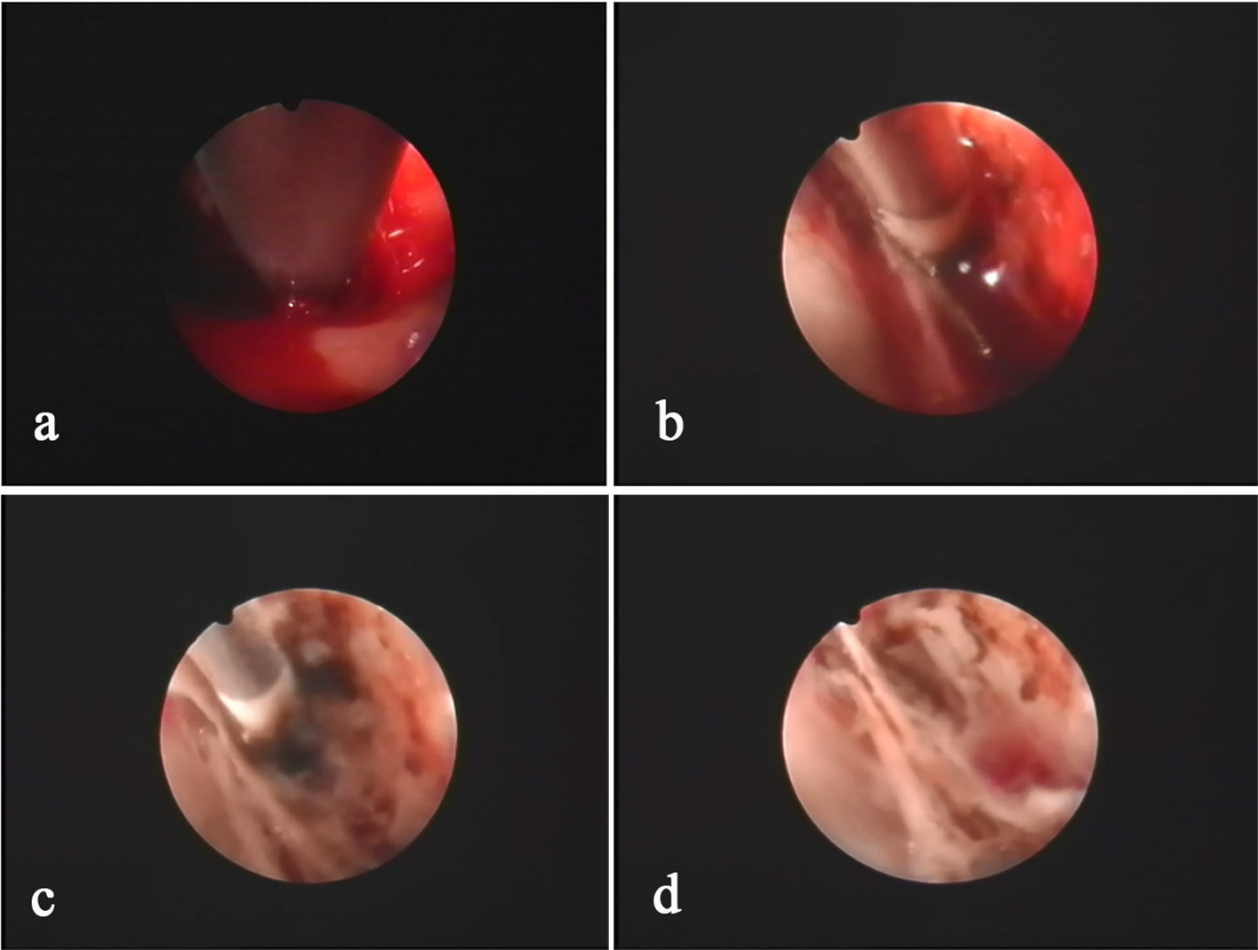
The hemorrhage sites inside the hematoma cavity are usually located at the folding point of the visceral layer and wall layer of the sac. The hemorrhage site **(a)** is identified after clearing the hematoma using an aspirator. Bleeding is precisely stopped using a specialized bipolar electrocoagulator on the endoscope **(b,c)**. After hemostasis, the hematoma cavity is washed with warm saline to confirm hemostasis **(d)**.

**Figure 4:**
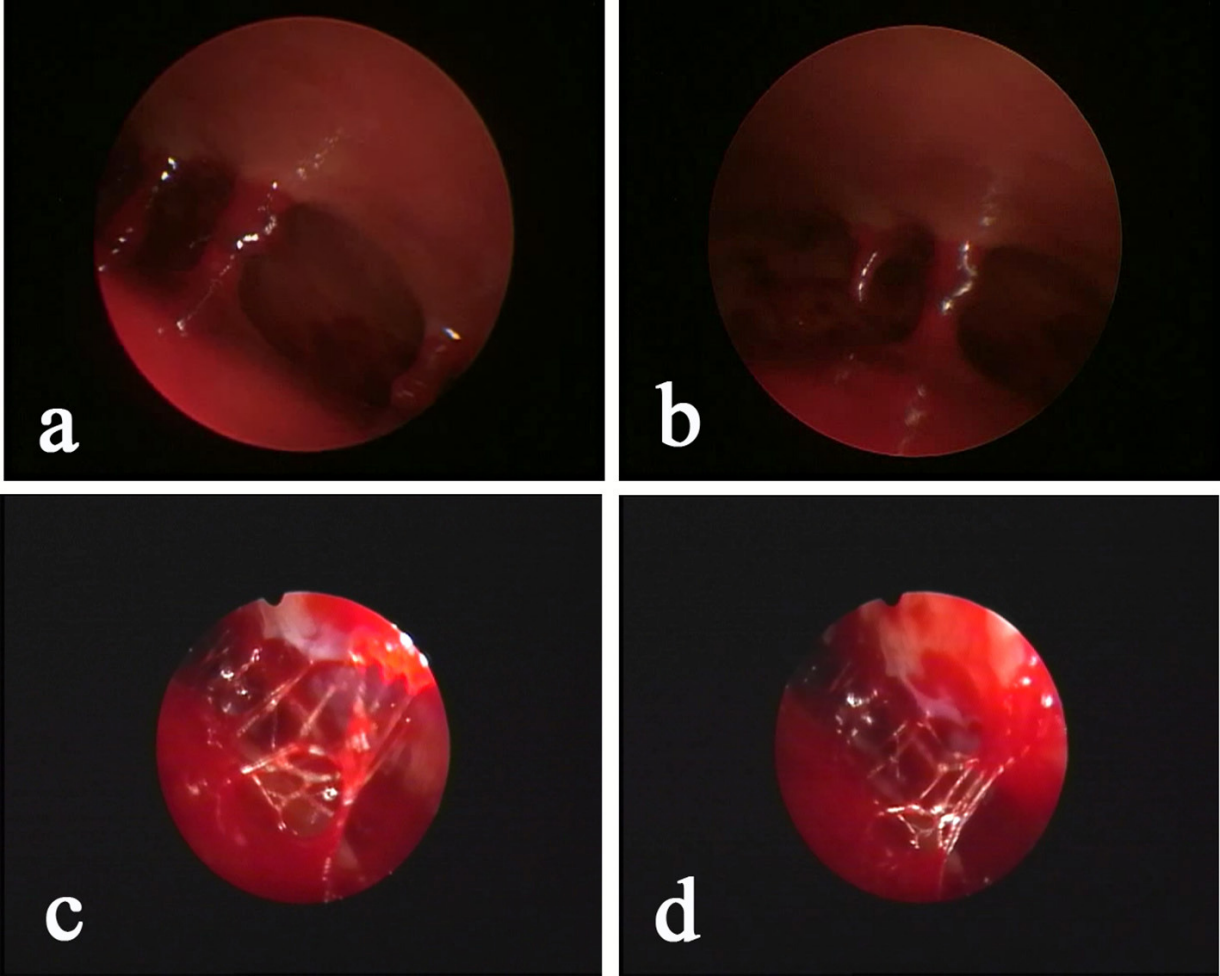
Neuroendoscopic observation of the hematoma with “separation,” as determined by pre-operative CT, shows that the “separation” is mainly a separation lock column **(a,b)** or separation strip **(c,d)** instead of a real closed septum. The relative separation is sufficient to block the blood clot in the chamber. Conventional BHD is not sufficient to completely drain the hematoma, but INET is able to remove the separation strip or separation lock column and visually clean the old blood clots.

During the surgery, all patients were under general anesthesia with tracheal intubation. The surgical incision was placed in the middle of the largest hematoma level, normally at the corner of the hematoma located 3–4 cm in front of the apical nodule. The incision, with a length of 4–5 cm, was parallel to the scalp vessels. The incision was either straight or “S” shaped (Figures 2a,b). After a hole was drilled in the skull, a bone flap (diameter, 2.0–3.0 cm) was cut using a milling cutter (Figure 2c). The wall layer of the hematoma sac was observed by a cross-shaped incision after lifting the dura mater (Figure 2d). The hematoma fluid was first extracted by puncture, and peripheral venous blood was taken simultaneously; both samples were sent for biochemical analyses, and the sac wall was sent for pathological examination. The endoscope was placed and was continuously flushed until clear. The hematoma fluid and old blood clots were removed through the endoscope working channel under alternating water and air environments, and the hemorrhage site was electrocoagulated to stop bleeding (Figure 3). The hematoma septal strips/columns were resected under the endoscope, and the blood clots in the septal chambers were removed (Figure 4). After cleaning the hematoma cavity, part of the hematoma wall and visceral capsule were removed from the bone window; the extent of the capsule resection was larger than 5.0 × 5.0 cm so that the hematoma cavity was fully connected to the subdural cavity (Video 1). After the surgery, the operation area was cleaned, and a drainage tube was visually placed in the subdural hematoma cavity. The small bone flap was fixed, the scalp was sutured layer by layer, and the skull was closed. Cranial CT was performed 24 h after surgery.

### Statistical Analysis and Sample Size Estimation

#### Statistical Analysis

The data were managed and analyzed using Stata 13.1 software. A multiple logistic regression model was established, and the factors that showed intergroup differences and surgical methods were included in the regression model to screen for independent factors that affected the outcome. The *t*-test was performed to determine differences in age, surgical duration, SDT placement duration, midline shift, and hematoma volume between two groups. The χ^2^ test and Fisher's exact probability test were used to compare the rates between two groups. The results for the quantitative data are expressed as the mean ± standard deviation. Endpoint event was defined as whether the subdural hematoma recurred by the 6 month follow-up. Patients who left the study, were lost to follow-up or died were exclude from the study. *P* < 0.05 was considered statistically significant.

#### Sample Size Estimation

The sample size was estimated based on the recurrence rate of the pilot experiment with α = 0.05 (according to the reference table, u0.05/1 = 1.96, bilateral), ß = 0.10 (according to the reference table, u0.10 = 1.282, bilateral) and power = 1-ß = 0.9. In the pilot experiment, the recurrence rate within 6 months after surgery for the INET group was Pe = 2.7%, the rate for the control group was Pc = 26.9%, and P = (Pe+Pc)/2 = 0.148, k = 1. The calculation indicated that at least 45 patients were required for each group.

Sample size estimation formula:

$$\begin{array}{l} N=\frac{{\left(u_{\alpha }+u_{\beta }\right)}^{2}\left(1+1/k\right)p\left(1-p\right)}{{\left(p_{e}-p_{c}\right)}^{2}} \end{array}$$

## Results

### Baseline Indicators and Clinical Indicators

The pre-operative baseline indicators, such as sex, average age, severity of midline shift, pre-operative hematoma volume, pre-operative Bender grade, and medical history (such as definitive trauma, anticoagulant drugs, hypertension, diabetes and stroke), did not differ significantly between the two groups (*p* > 0.05). However, the proportion of patients at an age of 65 years or younger (20.0 vs. 43.6%, *p* = 0.03) and the proportion of patients with unilateral subdural hematoma (91.4 vs. 69.2%, *p* = 0.02) differed significantly between the two groups (Table 1). The surgical incision length [(4.0–5.0) cm vs. (3.0–3.5) cm] and surgery duration for the INET group were significantly longer than those for the control group [(60.4 ± 10.6) min vs. (44.1 ± 9.8) min, *p* = 0.00], but the SDT placement duration [(2.1 ± 0.6) d vs. (3.9 ± 0.7) d, *p* = 0.00] and the hematoma recurrence rate at the 6 month follow-up (4.4 vs. 24.5%, *p* = 0.00) for the INET group were significantly lower than those for the control group. The intracranial infection rate (4.4 vs. 10.2%, *p* = 0.50) and the overall Bender grade effective rate at the 1 month follow-up (95.6 vs. 87.8%, *p* = 0.32) did not differ significantly between the two groups, but the proportion of patients who recovered to Bender grade 0 with no symptoms was significantly higher in the INET group than that in the control group (86.7 vs. 67.3%, *p* = 0.03) (Table 2).

**Table 2:** Comparison of intraoperative and post-operative clinical indicators between the INET and control groups.

**Clinical indicator**	**INET group** ** (*n* = 45)**	**Control group** ** (*n* = 49)**	***p*-value**
Surgery duration (min)	60.4 ± 10.6	44.1 ± 9.8	0.00
SDT placement duration (d)	2.1 ± 0.6	3.9 ± 0.7	0.00
Intracranial infection rate	2 (4.4%)	5 (10.2%)	0.50
Recurrence rate (6 months)	2 (4.4%)	12 (24.5%)	0.00
Overall effective rate	95.6%	87.8%	0.32
**Bender grade (1 month)**			
Grade 0	39 (86.7%)	33 (67.3%)	0.03
Grade I	5 (11.1%)	11 (22.4%)	0.14
Grade II	1 (2.2%)	5 (10.2%)	0.25

*Continuous variables are presented as the mean ± standard deviation, and categorical variables are presented as count (percentage)*.

### Biochemical and Pathological Examination Indicators

**Figure 5:**
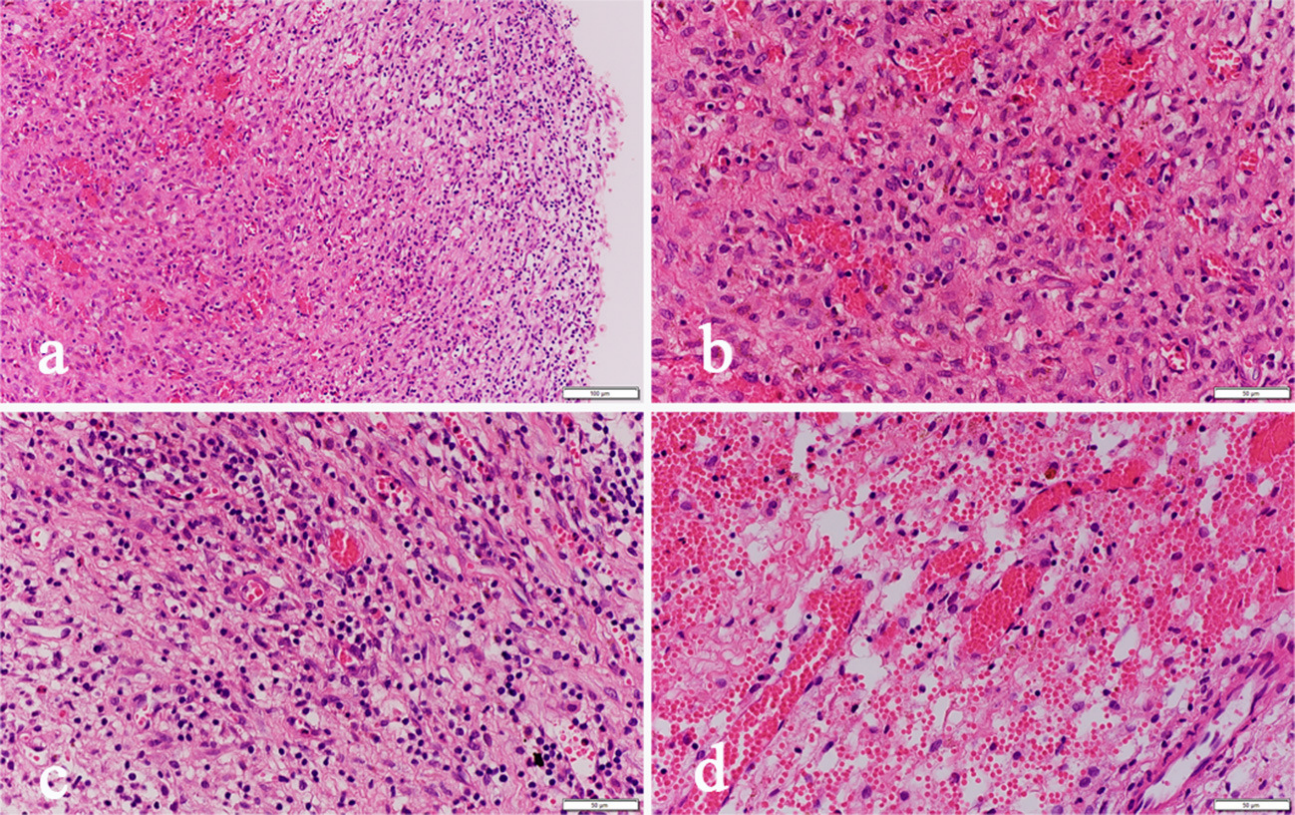
Pathological sections of the hematoma capsule: **(a)** HE-02-20X shows visible hemorrhage, vascular hyperplasia and inflammatory cell infiltration inside the capsule. **(b)** HE-03-40X shows visible hemorrhage and fibrovascular hyperplasia inside the capsule. **(c)** HE-04-40X shows visible inflammatory cell infiltration. **(d)** HE-05-40X shows visible neovascular rupture and bleeding.

Simultaneous examination of the hematoma fluid and peripheral venous blood showed that the osmotic pressure did not differ significantly between the two groups [(296.7 ± 10.3) mOsm/kg vs. (291.5 ± 12.4) mOsm/kg, *p* = 0.68]. The level of the proinflammatory cytokine interleukin 6 (IL-6) [(58.6 ± 14.6) pg/ml vs. (3365.8 ± 863.7) pg/ml, *p* = 0.00] and the content of D-dimer [(2044.5 ± 218.3) ng/ml vs. (1244236.8 ± 152545.6) ng/ml, *p* = 0.00], which reflects the local severity of fibrinolysis in the hematoma, were significantly higher than those in the peripheral venous blood (Table 3). Pathological examination of the hematoma wall capsule indicated angiogenesis inside the capsule that was accompanied with mucus-like changes and rupture of the newly generated vessels resulting in hemorrhage. Neutrophil and lymphocytic infiltration and fibrous connective tissue hyperplasia were observed in the hematoma capsule (Figure 5).

**Table 3:** Comparison of test indicators between subdural hematoma fluid and peripheral venous blood.

**Test indicator**	**Peripheral venous blood** ** (*n* = 94)**	**Hematoma fluid** ** (*n* = 94)**	***p*-value**
Osmotic pressure (mOsm/kg)	291.5 ± 12.4	296.7 ± 10.3	0.68
IL-6 (pg/ml)	58.6 ± 14.6	3365.8 ± 863.7	0.00
D-dimer (ng/ml)	2044.5 ± 218.3	1244236.8 ± 152545.6	0.00

### Multiple Logistic Regression Analysis

Logistic regression analysis showed that INET surgery [odds ratio (OR) 3.71, 95% confidence interval (CI) 1.31–9.62, *p* = 0.02], age of 65 years or younger (OR 1.51, 95% CI 1.05–2.87, *p* = 0.03) and unilateral subdural hematoma (OR 1.76, 95% CI 1.05–3.41, *p* = 0.02) were independent factors that reduced the post-operative recurrence rate (Table 4).

**Table 4:** Multiple logistic regression analysis results of the risk factors associated with post-operative hematoma recurrence.

**Risk factor**	**OR**	**95% CI**	***p*-value**
INET application (No)	Reference		
INET application (Yes)	3.71	1.31–9.62	0.02
Age>65 years	Reference		
Age ≤65 years	1.51	1.05–2.87	0.03
Bilateral subdural hematoma	Reference		
Unilateral subdural hematoma	1.76	1.05–3.41	0.02

*OR, odds ratio; CI, confidence interval; P-value of Wald test*.

## Discussion

Brain atrophy in middle-aged and elderly individuals in a natural state leads to expansion of the subarachnoid space and increased brain tissue activity, and the bridging veins become full and relatively prolonged; therefore, mild craniocerebral trauma is sufficient to cause drainage of the convex surface of the brain to the subdural bridging veins or rupture of the small veins adjacent to the sagittal sinus, leading to subdural hematoma (13). Surgery is the main treatment for cSDH, and common surgical methods include BHD and craniotomy. Currently, BHD is the main method and uses single or double holes. Some clinicians also inject urokinase into the hematoma cavity to accelerate blood clot efflux (2, 14). However, the recurrence rate after BHD is high in patients with subacute-chronic and chronic septal subdural hematomas. Our study showed that the hematoma recurrence rate within 6 months after surgery in the control group was as high as 24.5%, which is similar to the data reported in previous studies (15–17). Recent studies (18–21) have found that atorvastatin can reduce the recurrence rate of cSDH. To prevent intervention from this factor, all patients in this study were treated with 20 mg of atorvastatin once daily for 3 months. One study (22) showed that stopping hemorrhage at a new bleeding point of a subacute-chronic subdural hematoma is an important factor affecting post-operative recurrence, which can be better achieved in endoscopic surgery. In this study, we also found during surgery that almost all cases had obvious fresh bleeding at the transition of the visceral layer and wall layer of the hematoma sac that required electrocoagulation for hemostasis (Figure 3). Additionally, incomplete hematoma clearance during surgery is also an important cause of recurrence of post-operative hematoma (23, 24). Fibrin degradation products in residual blood clots can form hematoma capsules, and in the wall layer capsules, small bold vessels continue to bleed so that the hematoma cavity expands constantly. In our study, pathological examination of the hematoma capsule in the INET group showed that there were many newly generated blood vessels in the capsule and that the vascular wall was accompanied by mucus-like changes, incomplete and partial rupture, and bleeding. The sac wall had mass inflammatory cell infiltration and fibrous connective tissue hyperplasia (Figure 5). During the INET surgery, we observed thin and soft old blood clots inside many of the separated cavities of the chronic septal subdural hematoma that needed to be completely cleared by opening the septal cavity under endoscope (Figure 4). Conventional BHD is unable to treat septal strips and columns; therefore, blood clots are not drained completely in a short period of time, which is one of the important factors causing subsequent recurrence. Examination of the hematoma fluid and peripheral venous blood showed that the osmotic pressure did not differ significantly between the two [(296.7 ± 10.3) mOsm/kg vs. (291.5 ± 12.4) mOsm/kg, *p* = 0.68], suggesting that osmotic pressure was not the main cause of increased hematoma among the patients in this study. However, we found that the proinflammatory cytokine IL-6 in the hematoma fluid was significantly higher than that in the peripheral venous blood [(3365.8 ± 863.7) pg/ml vs. (58.6 ± 14.6) pg/ml, *p* = 0.00]. IL-6 can increase the gap between vascular endothelial cells and thus increase the permeability of blood vessels, suggesting that the inflammatory response is an important factor that leads to hematoma growth and recurrence (25–27). Additionally, our study also showed that the D-dimer level in the subdural hematoma fluid was significantly higher than that in the peripheral venous blood [(1244236.8 ± 152545.6) ng/ml vs. (2044.5 ± 218.3) ng/ml, *p* = 0.00], suggesting that local fibrinolysis in the hematoma cavity was hyperactive and continuous hyperactivation of fibrinolysis could cause recurrent bleeding of the hematoma sac wall, which is also an important factor that leads to subdural hematoma growth and recurrence (28, 29).

The INET used in this study can completely clear old blood inside the hematoma cavity and precisely stop bleeding at the bleeding sites. INET can also remove the separation strips/columns formed inside the hematoma cavity after electrocautery so that the hematoma cavity is fully connected, which helps adequate drainage. We did not find an intact septum in the hematoma of patients with septal subdural hematoma, as indicated in the preoperative CT imaging. The separations shown in the imaging were mainly separation strips/columns observed under endoscopy (Figure 4), but these relatively incomplete separations were able to prevent sufficient drainage of the blood clots inside the septal cavities through BHD. During the treatment of cSDH using neuroendoscopy, Shiomi et al. (30) found that a trabecular structure was present in 65% of the hematoma cavities and that 30% of the patients had blood clots inside the hematoma cavities. Therefore, removing the separation strips and columns under endoscopy can remove the support from the hematoma cavities and can thoroughly clear blood clots. Sufficient drainage is an important means to prevent recurrence. There are numerous blood clots inside subacute-chronic mixed subdural hematomas, and after drainage and decompression, hematomas are prone to bleeding again. As a result, the effect of simple BHD is poor. In contrast, INET can fully clear blood clots and stop bleeding at fresh bleeding sites by electrocoagulation (Figure 3). During surgery, the flexible compatibility of INET in both water and air environments is an advantage. Hemostasis of large bleeding sites can be achieved under an air environment, and small bleeding sites can be effectively stopped in a water environment by continuous flushing with 39°C saline. Our study found that the hematoma recurrence rate at the 6 month follow-up was 4.4% in the INET group, which was significantly lower than that in the control group. The latest report of recurrence rate using soft neuroendoscopic techniques to treat chronic subdural hematoma was 5.33% during 0.5–8 years of follow-up, which was similar to ours (31). All the recurrent cases were patients with bilateral subacute-chronic mixed subdural hematomas. Our study found that the structural characteristics of the cSDH cavity are as follows: (1) The bleeding site inside the hematoma cavity is often at the wall layer, especially at the fold between the wall layer and the visceral layer close to the dura mater, and the main root of the fiber strip or column is also at the wall layer. (2) Fiber strings are first formed inside the cavity, followed by septal columns and the strips/columns divide the hematoma into multiple chambers with different sizes. (3) The visceral layer of the hematoma wall rarely contains any blood vessels, and it is significantly thinner than the wall layer. The hematoma visceral layer in a few cases is absent under neuroendoscopy, and the sac wall visceral layer capsule is undetectable. (4) Normally, the space between the visceral capsule of the hematoma and the arachnoid membrane is easily separated, and there is only a little adhesion. After opening the visceral capsule, the neuroendoscope can enter the space for exploration in the water environment (Video 2). The key points of the INET surgery designed based on the above characteristics include the following. (1) The blood in the hematoma cavity is completely removed visually and the separation strips and columns inside the hematoma cavity can be removed with the help of simple instruments. Precise electrocoagulation can be performed at the bleeding sites inside the hematoma cavity to stop bleeding, especially at the fold between the visceral and wall layers. (2) Part of the hematoma cavity wall layer and visceral lay capsule are resected during the surgery. In this study, the resection range of the capsule in all patients was more than 5 cm in diameter to ensure sufficient connection between the hematoma cavity and the subdural space. The hematoma cavity was completely removed and washed before resection, and harmful substances, such as blood clots, eosinophils, fibrinogen, and inflammatory factors, were cleared to prevent a sterile inflammatory reaction in the subdural space. The follow-up data indicated that the overall effective rates for Bender grade at the 1 month follow-up did not differ significantly between the two groups (95.6 vs. 87.8%, *p* = 0.32), but the proportion of patients who recovered to Bender grade 0 with no symptoms was significantly higher in the INET group than in the control group (86.7 vs. 67.3%, *p* = 0.03), suggesting that INET treatment is more effective in alleviating clinical symptoms and improving neurological functions. Although the intracranial infection rate did not differ significantly between the two groups, the SDT placement time of the five patients with intracranial infection in the control group all exceeded 5 days. The low efficiency of simple BHD and the long duration of drainage were important causative factors of intracranial infection. Multiple logistic regression analysis showed that INET surgery (OR 3.71, 95% CI 1.31–9.62, *p* = 0.02), age of 65 years or younger (OR 1.51, 95% CI 1.05–2.87, *p* = 0.03) and unilateral subdural hematoma (OR 1.76, 95% CI 1.05–3.41, *p* = 0.02) were independent factors that reduced the post-operative recurrence rate (Table 4). Because the factors age and bleeding sites cannot be changed, our findings further show the value of INET in clinical application.

## Conclusions

In summary, the growth and recurrence of subacute-chronic and chronic septal subdural hematomas are complicated processes. Our study suggests that the inflammatory response in the hematoma cavity, angiogenesis in the hematoma capsule, and local hyperactivation of fibrinolysis may be associated with the growth and recurrence of subdural hematoma. Therefore, post-operative targeted drug treatments, such as glucocorticoids, anti-angiogenic drugs, and anti-fibrinolytic drugs, may improve the prognosis of patients, but this conclusion requires confirmation by further clinical studies. At present, surgery is the key treatment for this type of disease. The INET used in this study can be freely switched between water and air environments, making full use of the advantages of the two surgical environments. Surgical instruments can be delivered through the 3.7 mm working channel of the endoscope, which makes the operation more convenient and faster and the localization of the bleeding sites more accurate, thus reducing the probability of accidental injury to brain tissue when placing the instruments. Compared to conventional methods, the INET treatment plan, which is designed based on the structure of the hematoma sac wall and hematoma fluid composition, has a low hematoma recurrence rate and high safety and can improve patient prognosis, making it an effective surgical method for the treatment of this type of disease.

## Limitations

The present study does have some limitations. First, it was a non-randomized concurrent control study conducted in two centers and this was liable to selection bias. Second, although strict inclusion/exclusion criteria were setted and statistical methods were used to control selection bias, we still couldn't avoid the occurrence of it. Third, the surgeons responsible for surgery are trained doctors, but differences in individual clinical skills are unavoidable. Therefore, the conclusions require confirmation in future multicenter randomized controlled trial and long-term follow up.

## Data Availability Statement

All datasets generated for this study are included in the article/Supplementary Material.

## Ethics Statement

The studies involving human participants were reviewed and approved by Shenzhen People's Hospital (LL-KT-2018245) and Nanfang hospital (NFEC-2015-034). The patients/participants provided their written informed consent to participate in this study.

## Author Contributions

YZ, BD, YP, and AS conceived and designed the whole experiments and manuscript. YZ, JH, JX, and XZ performed the statistical analysis and interpreted the data. JL and PL contributed to the literature research. HW and WL acquired the data. All authors read and approved the final manuscript.

### Conflict of Interest

The authors declare that the research was conducted in the absence of any commercial or financial relationships that could be construed as a potential conflict of interest.

## Supplementary Material

Video 1 After clearing the old blood in the hematoma cavity and confirmation of hemostasis, the visceral capsule of the hematoma cavity wall is removed by INET using the grasping forceps under the water environment; the resection range is larger than 5 cm.

Video 2 After opening the visceral layer of the hematoma capsule, INET was used to enter the hematoma visceral capsule and the arachnoid space for exploration in the water environment. The septum is easily peeled off with only a little adhesion band remaining.
