# Supplementary material for: A Clinical Study of the Intra-Neuroendoscopic Technique for the Treatment of Subacute-Chronic and Chronic Septal Subdural Hematoma
Source: Front Neurol. 2020 Jan 17;10:1408. doi: 10.3389/fneur.2019.01408 (PMC6979069; doi:10.3389/fneur.2019.01408)
Supplement: Supplementary file 1 [file Data_Sheet_3.DOCX]

| Project name | Multi-center clinical controlled study of intra-neuroendoscopic technique to treat intracranial hematoma |
| --- | --- |
| department | Emergency department |
| Project manager | BO DU ,Yujuan ZHANG,Aijun Shan et al |
| Project category | Shenzhen Key Clinical Research Project |
| Project time | 2018.2-2021.6 |
| Ethical approval | Shenzhen Health Department |
| List of documents for review | Ethics Review Report |
| Review mode | Quick review |
| Examiners | Xiaofang Yu, Wei Zhang, Jianhong Wang et al. |
| Final decesion | Agree |
| Notation | 1. Continued clinical controlled studies based on previous research results. 2. Intra-neuroendoscopic technique (INET) and traditional drilling drainage and craniotomy for hematoma in brain parenchyma. 3. Comparison of intra-neuroendoscopic technique (INET) and traditional drilling drainage for ventricular system hemorrhage. 4. Comparison of intra-neuroendoscopic technique (INET) and traditional drilling drainage for for the treatment of subacute-chronic and chronic septal subdural hematoma. 5. Valid until June 30^th^,2021 |
| Ethics committee | Ethics committee of Shenzhen People’s Hospital |
| Chairman | Xiaofang-Yu |
