# Supplementary material for: A Clinical Study of the Intra-Neuroendoscopic Technique for the Treatment of Subacute-Chronic and Chronic Septal Subdural Hematoma
Source: Front Neurol. 2020 Jan 17;10:1408. doi: 10.3389/fneur.2019.01408 (PMC6979069; doi:10.3389/fneur.2019.01408)
Supplement: Supplementary file 2 [file Data_Sheet_4.DOCX]

| Project name | Clinical comparative study of two methods for clearing intracranial hematoma |
| --- | --- |
| department | Neurosurgery department |
| Project manager | Yuping Peng,BO DU et al |
| Project time | 2015.5.21-2016.5.21 (Can be extended for 3 years) |
| Ethical approval | Shenzhen Health Department |
| List of documents for review | Ethics Review Report |
| Review modes | Full board review |
| EC members | Xn Zhang,Zhigang Guo,Shaolin Shen,Ping Jiang,Yabing Guo,Libo Li,Hongzhen Zhou,Shiting Liu,Jinhai Yan, |
| Final decesion | Agree |
| Notation | 1. Continued clinical controlled studies based on previous research results. 2. Intra-neuroendoscopic technique (INET) and traditional drilling drainage and craniotomy for hematoma in brain parenchyma. 3. Comparison of intra-neuroendoscopic technique (INET) and traditional drilling drainage for ventricular system hemorrhage. 4. Comparison of intra-neuroendoscopic technique (INET) and traditional drilling drainage for for the treatment of subacute-chronic and chronic septal subdural hematoma. |
| Ethics committee | Ethics committee of Nanfang Hospital |
| Chairman | Xun Zhang |
